# Statistical Mechanics of the Human Placenta: A Stationary State of a Near-Equilibrium System in a Linear Regime

**DOI:** 10.1371/journal.pone.0142471

**Published:** 2015-11-16

**Authors:** Yves Lecarpentier, Victor Claes, Jean-Louis Hébert, Xénophon Krokidis, François-Xavier Blanc, Francine Michel, Oumar Timbely

**Affiliations:** 1 Centre de Recherche Clinique, Centre Hospitalier Régional de Meaux, Meaux, France; 2 Department of Pharmaceutical Sciences, University of Antwerp, Wilrijk, Belgium; 3 Institut de Cardiologie, Hôpital de la Pitié-Salpêtrière, Assistance Publique-Hôpitaux de Paris, Paris, France; 4 Service de Pneumologie, Centre Hospitalo-Universitaire de Nantes, Nantes, France; 5 Service de Gynécologie-Obstétrique, Centre Hospitalier Régional de Meaux, Meaux, France; PreTel Inc, UNITED STATES

## Abstract

All near-equilibrium systems under linear regime evolve to stationary states in which there is constant entropy production rate. In an open chemical system that exchanges matter and energy with the exterior, we can identify both the energy and entropy flows associated with the exchange of matter and energy. This can be achieved by applying statistical mechanics (SM), which links the microscopic properties of a system to its bulk properties. In the case of contractile tissues such as human placenta, Huxley’s equations offer a phenomenological formalism for applying SM. SM was investigated in human placental stem villi (PSV) (n = 40). PSV were stimulated by means of KCl exposure (n = 20) and tetanic electrical stimulation (n = 20). This made it possible to determine statistical entropy (S), internal energy (E), affinity (A), thermodynamic force (A / T) (T: temperature), thermodynamic flow (v) and entropy production rate (A / T x v). We found that PSV operated near equilibrium, i.e., A ≺≺ 2500 J/mol and in a stationary linear regime, i.e., (A / T) varied linearly with v. As v was dramatically low, entropy production rate which quantified irreversibility of chemical processes appeared to be the lowest ever observed in any contractile system.

## Introduction

Human placental stem villi (PSV) have been shown to possess contractile properties [1,2] and the kinetics of myosin crossbridge (CB) molecular motors inside the extravascular part of PSV are dramatically slow [3]. A phenomenological approach of the thermodynamic behavior of myosin CBs is provided by the A. Huxley theory [4] which allows to determine kinetics and probabilities of several steps of the ATP-ADP-Pi-acto-myosin cycle [5]. A. Huxley’s equations link kinetics of CB molecular motors to mechanics of the whole contractile tissue. On the other hand, statistical mechanics (SM) is devoted to the study of physical properties of macroscopic systems consisting of a very large number of molecules. The huge number of CBs involved in contractile processes confers the necessary ground for applying SM. From a thermodynamic point of view, contractile tissues are open living systems exchanging energy and matter with their surroundings. They are not in thermodynamic equilibrium and can operate either near or far away from the thermodynamic equilibrium [6]. The goal of our study was to apply SM to human PSV by using the grand canonical ensemble which represents a general and useful method for studying complex open systems. SM combined with the A. Huxley formalism made it possible to determine statistical entropy (S), internal energy (E), chemical affinity (A), thermodynamic force (A / T) (T: temperature), thermodynamic flow (v), entropy production rate (A / T x v) in human PSV [4,7,8]. We observed that human PSV operated under linear regime in a near-equilibrium stationary state. Thermodynamic flow was particularly low in human PSV, which conferred a dramatically low entropy production rate. In the Discussion section, results obtained in human PSV were compared to those previously published by using the same formalism [9,10]. Human PSV mechanical and biochemical properties were compared with their counterparts in currently used smooth and striated (heart and skeletal) mammalian muscles [11–13].

## Materials and Methods

### Ethics Statement

Placenta (n = 40) were obtained from pregnant women undergoing delivery at the maternity of Meaux hospital, Meaux. Patients gave informed written consent. The study was approved by the « Comité Consultatif sur le Traitement de l’Information en matière de Recherche dans le domaine de la Santé » 2012.181, the « Local Ethical Committee », 2011, 206, the « Direction Générale pour la Recherche et l’Innovation » and the Direction of the Meaux Hospital, Meaux. All pregnancies were normal without complications. Several exclusion criteria were retained: delivery before 37 weeks of gestational age, newborn with birth weight below the 10^th^ percentile [14], pre-eclampsia, gestational diabetes, congenital malformations and chromosomal abnormalities.

### Experimental Protocol

Human PSV were carefully cut from the middle part of a cotyledon (resting length Lo was 10.2±2.6 mm and diameter was 1.5±0.3 mm) belonging to the type 1 category of the Demir classification [15]. PSV were horizontally disposed in a bath chamber and were held by means of a spring clip attached to the lever arm of a homemade electromagnetic system [16]. A basal tone of about 1 mN.mm^-2^ was imposed to the PSV in such a way it induced neither shortening nor lengthening of the PSV. The bath chamber contained a Krebs-Henseleit solution (in mM): 118 NaCl, 4.7 KCl, 1.2 MgSO_4_.7 H_2_O, 1.1 KH_2_PO_4_, 24 NaHCO_3_, 2.5 CaCl_2_.6H_2_O and 4.5 glucose, maintained at 22°C, pH 7.4, and bubbled with 95% O_2_-5% CO_2_. Stimulation of human PSV was induced by either electrical tetanus (tetanus group; n = 20) (train period: 5s; train duration: 2s; stimulus frequency: 100 Hz; stimulus duration: 5ms) or after exposure to KCl (KCl group; n = 20) at 0.05 M concentration.

### Electromagnetic Lever System

The electromagnetic device was previously described in details [16]. A precision source delivered a current through the coil and hence determined the force at the tip of the lever. This force could be set by decade switches in calibrated steps of 0.01, 0.1,1 and 10 mN, up to a total of 20 mN. The displacement of the lever was measured by means of an opto-electronic transducer. A light-emitting diode (LED) was mounted in such a way that a diaphragm on the lever modulated the light falling on the photodiode. The photodiode current was converted into a voltage and filtered with an active third order low pass filter. The length range was 1000 μm resulting in an output voltage of 10 V full scale. Force carried by the PSV was measured with unilateral feedback techniques. Maximum range was 10 V, corresponding to a load of 20 mN at the lever tip. The electrical stimulus was provided through two platinum electrodes disposed along both sides of the PSV.

### Hyperbolic Tension-Velocity (T-V) Relationship

The load level was progressively decremented by successive steps of 0.1mN from isometric tension until zero-load. At each afterload level, PSV shortened at isotonic tension level (T) and constant velocity (V) [3]. The T-V relationship was constructed from the velocity (V) of 7 to 10 isotonic contractions, versus the isotonic load level normalized per cross-sectional area. The T-V relationship was fitted by means of a hyperbola according to the Hill equation (T+a)(V+b) = (To+a)b, where–“a” and–“b” are the asymptotes of the hyperbola [17]. The G curvature of Hill's equation is equal to To / a = Vo / b, where To is the peak isometric tension, and Vo the maximum unloaded shortening velocity. Asymptotes–“a” and–“b” were expressed in mN/mm^2^ and in Lo/s, respectively. G is dimensionless.

### A. Huxley Equations

**Fig 1:**
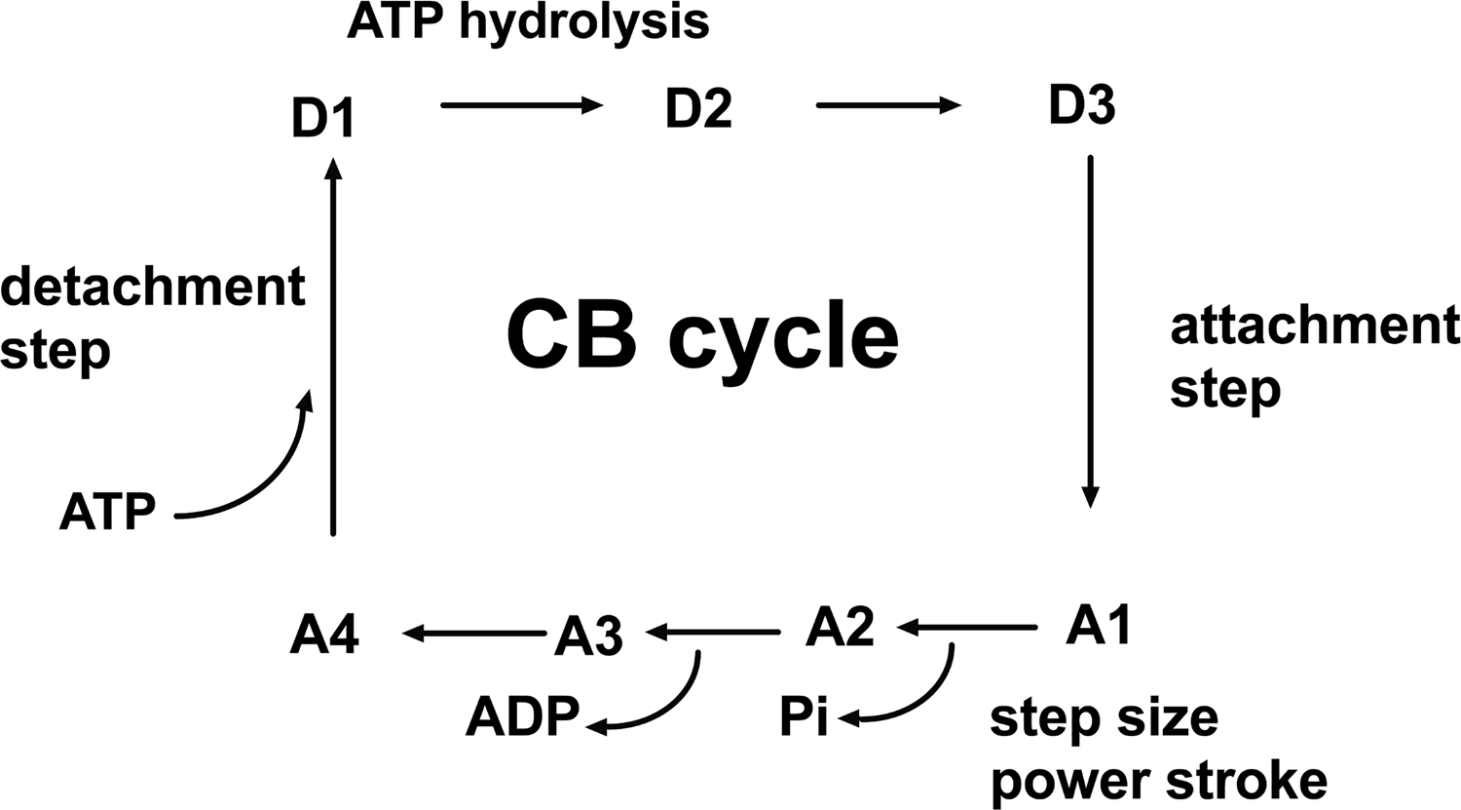
The cycle of ATP-ADP-Pi- acto-myosin. The CB cycle was subdivided into seven different conformational states, with three detached states (D1, D2, and D3) and four attached states (A1, A2, A3 and A4). Transition A4 → D1 was the ATP binding step; D1 was the first detached step. CB detachment occurred when ATP bound to the actin (A)—myosin (M) complex (AM). The rate constant for detachment was g_2_; AM + ATP → A + M-ATP. Transition D1 → D2 was the ATP hydrolysis step; M-ATP → M-ADP-Pi. Transition D2 → D3 was M-ADP-Pi → M*-ADP-Pi. D3 was the step having the highest probability. Transition D3 → A1 was the weakly bound attachment state: the myosin head (M*-ADP-Pi) weakly bound to A. The rate constant for attachment was f_1_; M*-ADP-Pi + A→ AM-ADP-Pi. Transition A1 → A2 was the power stroke or strongly bound state which was triggered by the Pi release: AM-ADP-Pi → AM-ADP + Pi. Transition A2 →A3 was the release of ADP: AM-ADP → AM + ADP. A4 was a transitory attached step (AM) having the lowest probability and preceeding the detachment step D1 induced by the attachment of a new ATP molecule.

The CB cycle was described in Fig 1. A. Huxley’s equations can be applied to striated and smooth muscles and human PSV [3,4]. They made it possible to calculate the peak rate constants for CB attachment (f_1_ in s ^-1^) and CB detachment (g_1_ and g_2_ in s ^-1^), catalytic constant (k_cat_ in s ^-1^), myosin content (mole number per liter in nM/L), and thermodynamic flow (v), i.e., maximum myosin ATPase activity under isometric conditions where v = k_cat_ x mole number per liter. Moreover, w is the maximum mechanical work of a single CB (w / e = 0.75) and e is the free energy required to split one ATP molecule [4]. According to A. Huxley’s theory, one ATP is split per CB cycle, the ATP standard free energy is nearby– 60 kJ /mol, thus the value used for e was equal to 10^−19^ J. The molecular step size h is defined by the translocation distance of the actin filament per ATP hydrolysis. In vivo double-head myosin displacement during a unitary interaction with actin was 10 nm [18]. The parameter l is the distance between successive actin sites with which any myosin site can combine with actin. According to in vivo conditions and A. Huxley’ s formalism (1 ≻≻ h) [4], the values of h and l were h = 10 nm and l = 28.6 nm which is close to the semi helicoidal turn of the actin filament. Calculations of f_1_, g_1_, g_2_ and k_cat_ were given by the following equations [9]:

$$\text{G}={\text{f}}_{1}/{\text{g}}_{1}$$

$${\text{g}}_{1}=(2\;\text{wb})/(\text{ehG})$$

$${\text{g}}_{2}=(2\;{\text{V}}_{\text{max}})/\text{h}$$

$${\text{k}}_{\text{cat}}=(\text{h}\;{\text{f}}_{1}\;{\text{g}}_{1})/\left[\left(2\text{l})({\text{f}}_{1}+{\text{g}}_{1}\right)\right]$$

### Statistical Mechanics (SM)

The grand canonical ensemble is a useful tool for applying SM to complex open systems such as living contractile tissues, and has been previously used [9,10]. The open contractile placental system (**S**) was in a container (**C**), both exchanging energy and matter. **S** was composed of all the cycling myosin CBs that could individually be found in one of the seven distinct states, either attached to or detached from actin, and bound or not to a nucleotide (ATP, ADP) or to Pi. **C** was composed of all the non-cycling myosin CBs, all the non-cycling actin molecules, and all the ATP and ADP and Pi which were not attached to the cycling myosin CBs. Actin, myosin CBs and small soluble molecules (i.e., ATP, ADP and inorganic phosphate Pi) can be exchanged between **S** and **C**, so that the number of cycling CBs may slightly fluctuate with the number of non cycling CBs becoming cycling CBs and vice versa. In the grand canonical ensemble, the average number of independent, non interacting cycling CBs within S was determined from A. Huxley’s equations [4] and was expressed in nM per L of placental tissue. The grand potential (Ψ) is linked to E, S, A and T according to the classic relationship Ψ = E−TS−A where A is the chemical affinity of the CB cycle, S the statistical entropy, E the internal energy, and T (Kelvin) the temperature of the system **S**. The method for determining the probability (Pr) of the seven (r = 7; 4 attached states and 3 detached states) conformational states of the CB cycle has been previously described [9] (Fig 1). Statistical entropy S is given by the formula S = −R∑ PrlnPr (R: gas constant). The molecular partition function is z = 1/Pmax (where Pmax is the highest probability PD3 of step D3 of the CB cycle). Briefly, for each of the seven different conformational states r (i.e., r = 0, 1, …,6) of the CB cycle, a given energy level E_r_ was individualized. The energy level E_r_ increased from E_0_ to E_6_. By convention, the lowest level (E_0_), which coincided with the ground state (gs), was equal to zero; thus Eo = Egs = 0. The most probable detached state was D3; this implies that Po = PD3 and consequently Eo = ED3 = 0. The Boltzmann distribution is given by the equation:

P_r_ = exp (−βE_r_)/∑ exp (−βE_r_), and by definition the molecular partition function is equal to z = ∑exp(−βE_r_), thus P_r_ = exp (−βE_r_)/z, where β = 1 / kT. As Eo = 0 consequently, Po = PD3 = 1/z.

Moreover, we have the thermodynamic equation: E−TS = −RT lnz

Thus E−TS = Ψ−A = −RT lnz = RT lnPmax = RT lnPD 3. Consequently E = RTlnPmax+TS. The CB cycle stopped when the thermodynamic flow ϖ of the system **S** tended towards zero. Under these conditions, A also tended towards zero. Thus E−TS tended towards Ψ. The extrapolation at v = 0 of the E−TS versus v relationship (i.e., the ordinate of this relationship) was equal to Ψ. Affinity A was calculated by the equation: A = Ψ−RT lnPD 3 and thermodynamic force was equal to A/T.

If A ≺≺ 2500 J/mol, a chemical system operates near-equilibrium. A near-equilibrium chemical system under linear regime evolves to a stationary state. This is achieved if the thermodynamic force A/T varies linearly with the thermodynamic flow v [19,20]. In open systems in their stationary state, entropy production rate (diS/dt) due to a chemical reaction is the product of thermodynamic force and thermodynamic flow: diS/dt = (A/T) x v [20,21].

## Results

**Fig 2:**
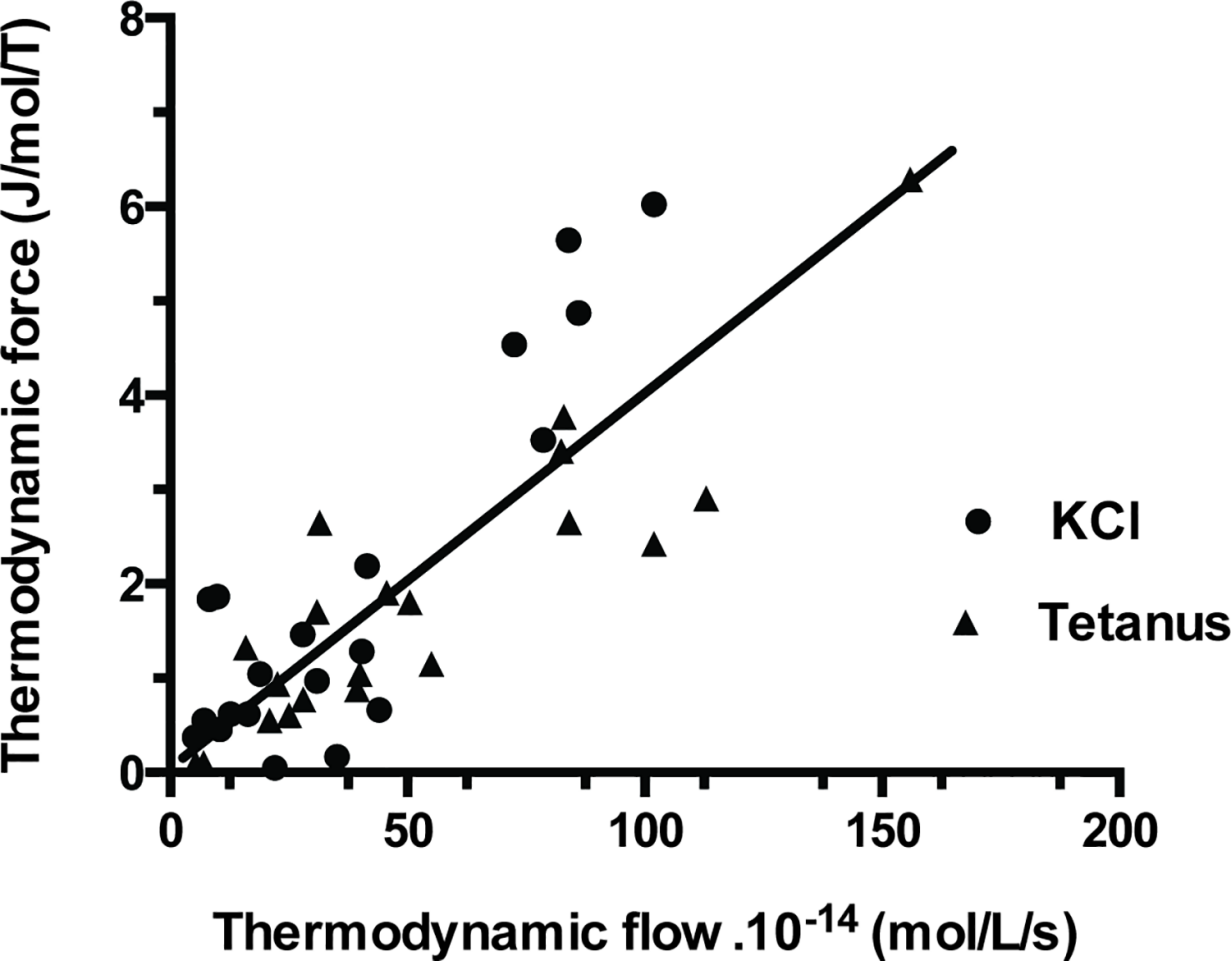
Thermodynamic force versus thermodynamic flow relationship. Thermodynamic force varied linearly with thermodynamic flow. The linear fit was thermodynamic force = 0.112 + (3.97. 10^-12^) thermodynamic flow; r = 0.84.

**Fig 3:**
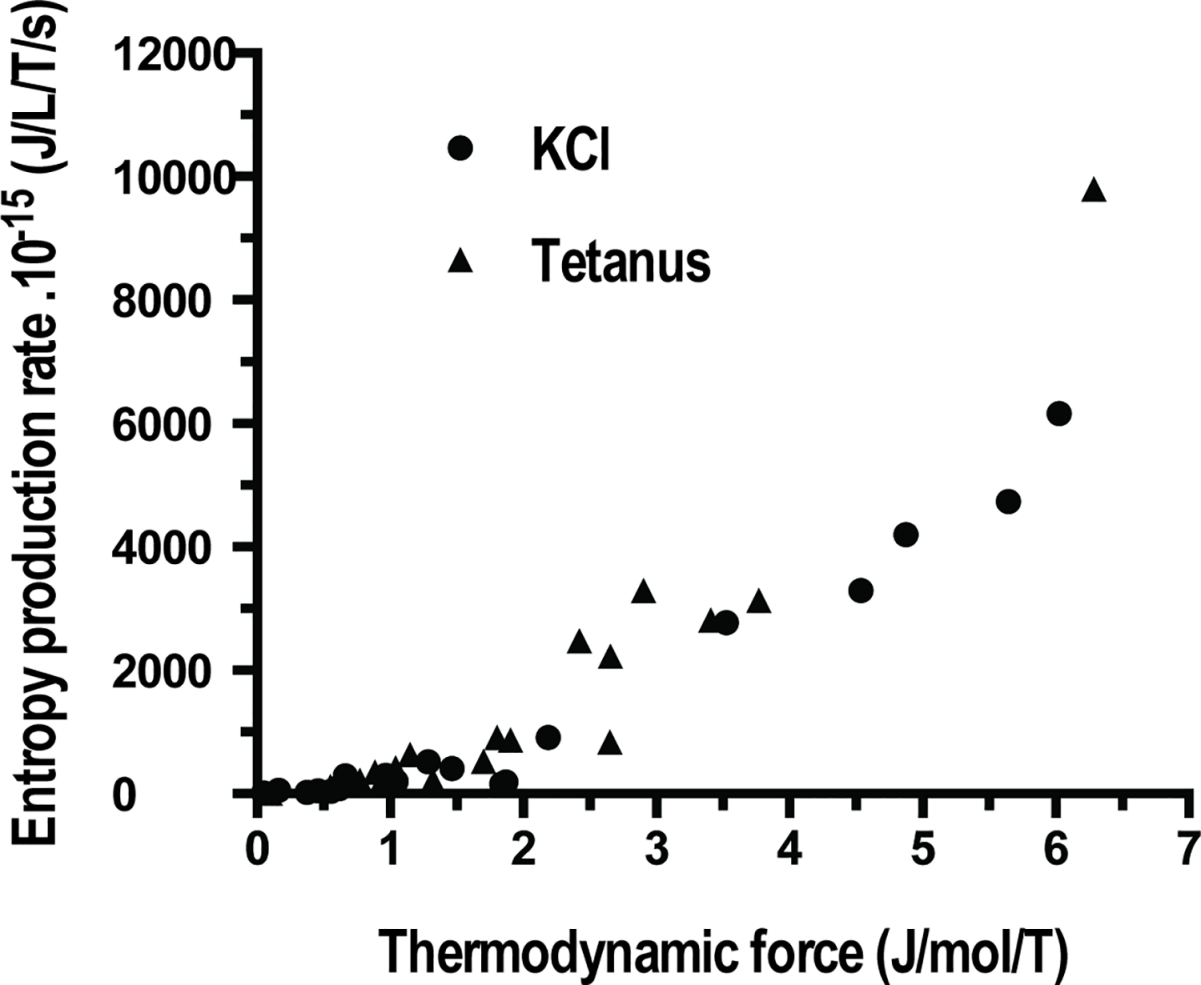
Entropy production rate versus thermodynamic force. There was a curvilinear relationship between entropy production rate and thermodynamic force.

**Fig 4:**
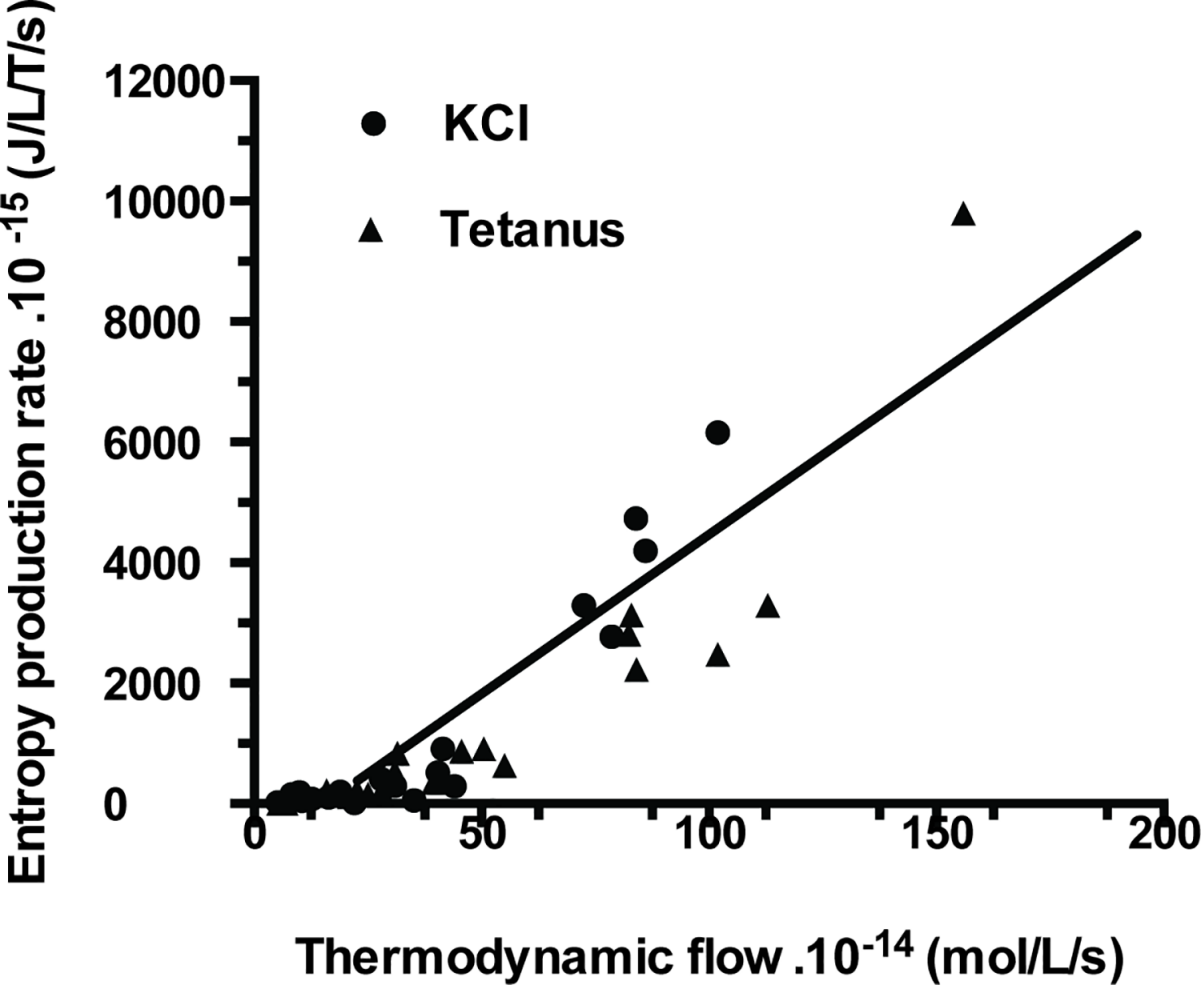
Entropy production rate versus thermodynamic flow relationship. Entropy production rate varied linearly with thermodynamic flow. The linear fit was entropy production rate = (- 9.9. 10^-13^) + 5.2 thermodynamic flow; r = 0.91.

**Fig 5:**
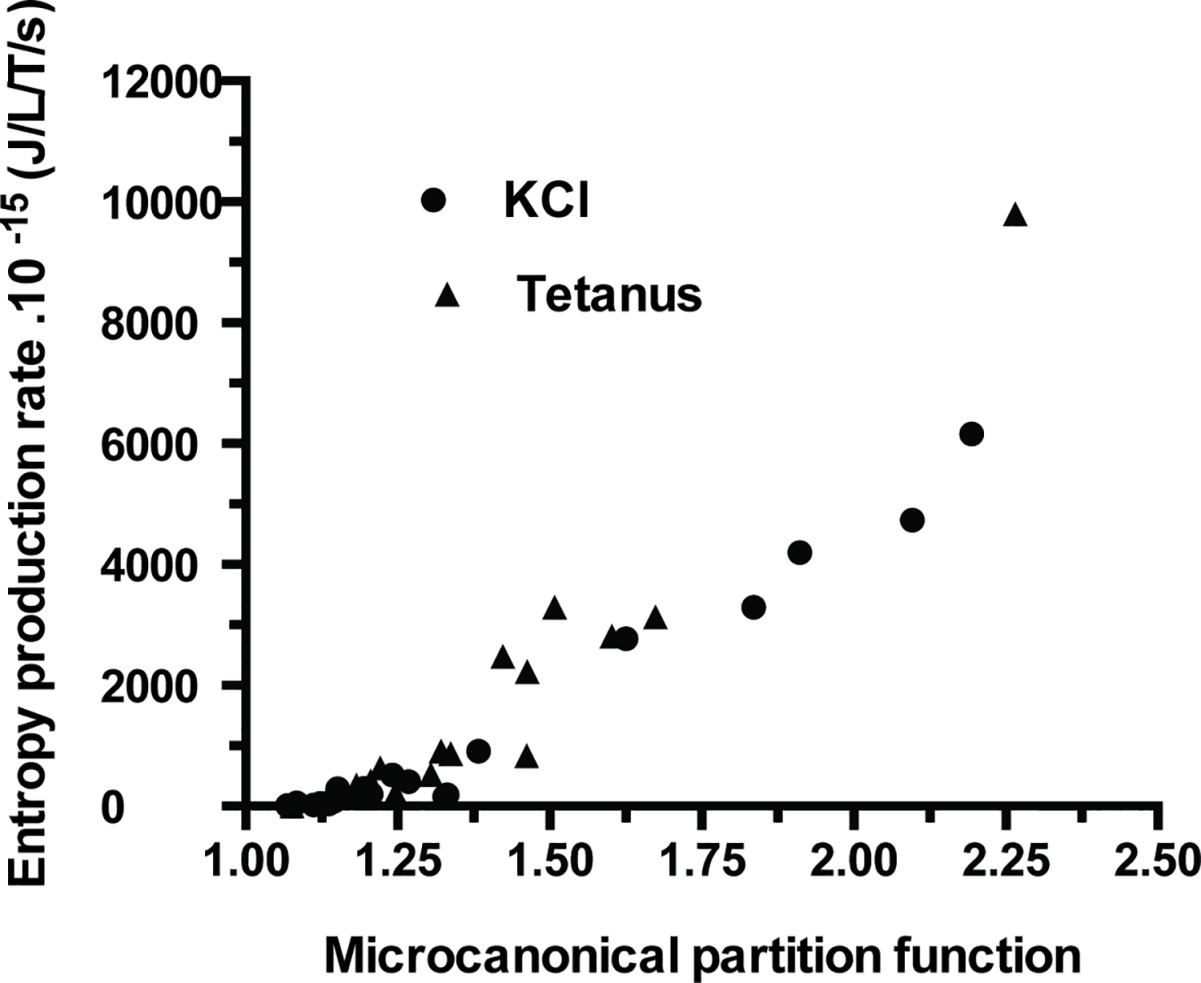
Entropy production rate versus microcanonical partition function. There was a curvilinear relationship between entropy production rate and microcanonical partition function.

**Fig 6:**
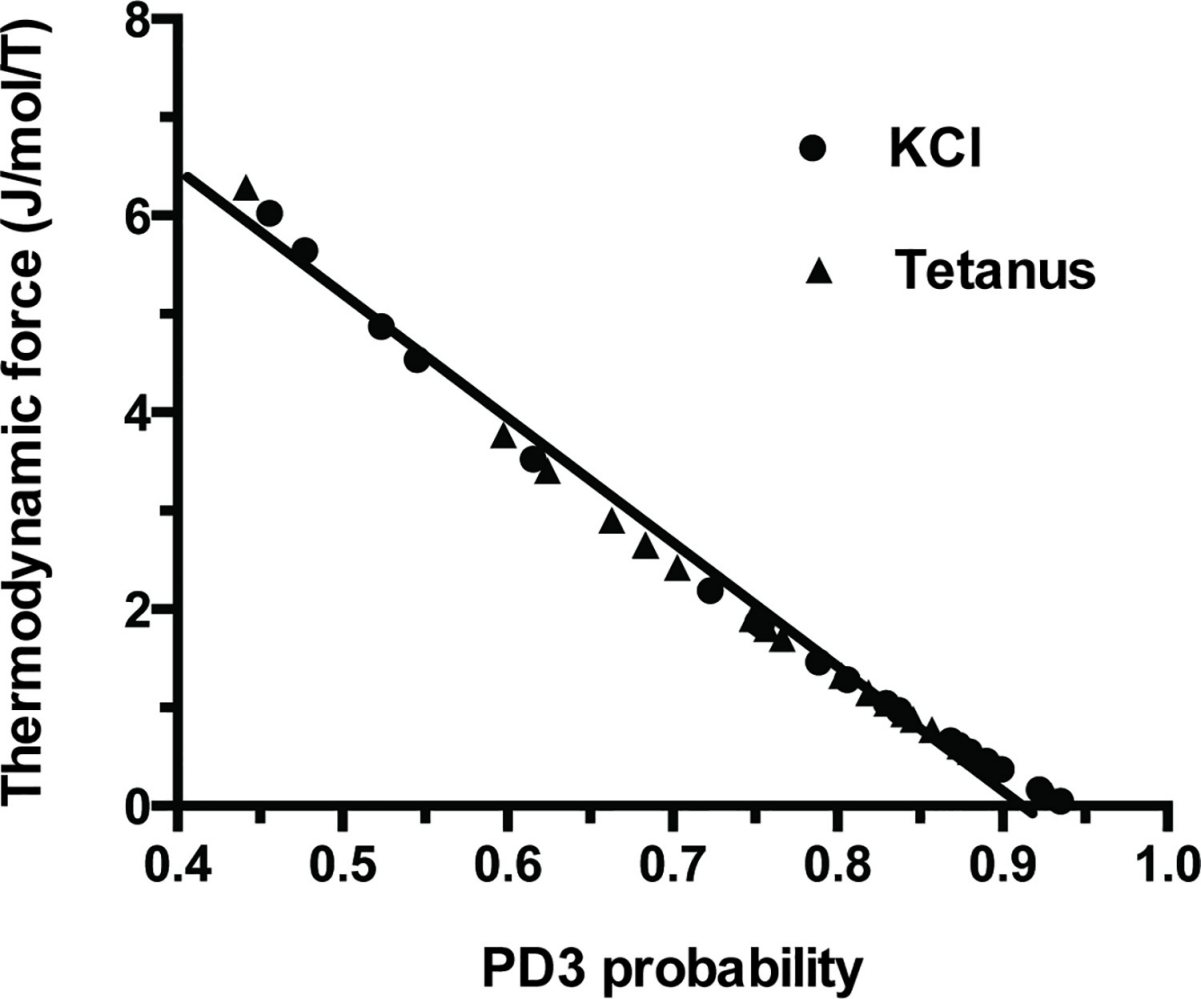
Thermodynamic force versus PD3 probability relationship. There was a negative linear relationship between thermodynamic force and PD3 probability. Thermodynamic force = 1.2–12.2 PD3; r = 0.99.

Affinity (A) of PSV was 573±562 J/mol (mean ± SD) in KCl and 549±441 J/mol in tetanus (Table 1; NS). Thus, PSV behaved near-equilibrium because A ≺≺ 2500 J/mol. Moreover, thermodynamic flow varied linearly with thermodynamic force (Fig 2). Thus, PSV operated in a stationary linear regime. Table 1 shows that the following thermodynamic parameters did not differ according to the stimulation mode either under tetanus or KCl, i.e., affinity, thermodynamic force, thermodynamic flow, entropy production rate, statistical entropy, internal energy, microcanonical partition function, catalytic constant and myosin content. Entropy production rate was dramatically low because of the very low value of the thermodynamic flow and it increased curvilinearly with thermodynamic force (Fig 3) and linearly with thermodynamic flow (Fig 4). Entropy production rate also increased curvilinearly with the microcanonical partition function (Fig 5). Thermodynamic force decreased linearly with the highest probability (PD3) (Fig 6).

**Table 1:** Thermodynamic parameters of human placental stem villi (PSV) under electrical tetanus and KCl [0.05M].

	Electrical tetanus; n = 20	KCl exposure; n = 20	p
Affinity	549±441	573±562	0.88
Thermo force	1.85±1.49	1.93±1.90	0.87
Thermo flow	5.1±3.9 (10^−13^)	3.8±3.0 (10^−13^)	0.21
EPR	1.45±2.25 (10^−12^)	1.22±1.89 (10^−12^)	0.72
Entropy	6.2±1.9	5.9±.2.1	0.71
Internal energy	1150±221	1052±.201	0.15
z	1.35±0.27	1.38±0.35	0.78
k_cat_	.004±.003	.002±.002	0.15
myosin content	.173±.090	.145±.048	0.20

Affinity of the CB cycle (J/mol); Thermodynamic force (J/mol/T); Thermodynamic flow (mol/L/s); EPR: Entropy production rate (J/T/L/s); S: Statistical entropy (J/mol/T); E: Internal energy (J/mol)); z: microcanonical partition function (dimensionless); k_cat_: catalytic constant (s^-1^); myosin content (nM/L). Values are means ± SD. For all parameters presented in Table 1, there were no statistical differences between tetanus and KCl exposure by applying the unpaired t test, as attested by the p-values.

## Discussion

The purpose of our study was to apply the formalism of statistical mechanics (SM) to human placental stem villi (PSV). SM made it possible to determine thermodynamic parameters such as statistical entropy, internal energy, chemical affinity, thermodynamic force, thermodynamic flow, entropy production rate of a given chemical system [6]. We found that human PSV was a near-equilibrium system which operated under linear regime in a stationary state. Thermodynamic flow appeared to be dramatically low. SM parameters of PSV (Table 1) were compared to those of striated (heart and skeletal) and smooth muscles previously published in the literature and according to a similar method (Table 2).

**Table 2:** Mechanical parameters of smooth and striated muscles and thermodynamic parameters previously published in the literature with the same method [9,10].

	Trachea	Heart	Soleus	EDL
Affinity	388±221	535±311	55±37	101±56
Thermo force	1.25±.71	1.77±1.03	.19±.13	.34±.19
Thermo flow	2.9±1.1(10^−9^)	2.8±1.7(10^−7^)	4.6±2.3(10^−9^)	4.6±1.9(10^−8^)
EPR	4.2±3.5(10^−9^)	6.5±6.6(10^−7^)	9.8±9.9(10^−9^)	1.7±1.4(10^−8^)
S	8.7±.8	9.3±.7	2.3±.5	4.5±.5
E	1646±56	1401±140	678±85	987±83
z	1.5±.1	1.8±.2	1.1±.1	1.2±.1
k_cat_	.4±.2	20.6±7.2	.9±.3	8.0±1.9
myosin content	7.1±1.7	12.5±4.5	4.9±1.2	5.6±1.5

Affinity of the CB cycle (J/mol); Thermodynamic force (J/mol/T); Thermodynamic flow (mol/L/s); EPR: Entropy production rate (J/T/L/s); S: Statistical entropy (J/mol/T); E: Internal energy (J/mol)); z: microcanonical partition function (dimensionless); k_cat_: catalytic constant (s^-1^); myosin content (nM/L). Values are means ± SD. EDL, Soleus and Trachea were stimulated under tetanic electrical stimulation and Heart under twitch electrical stimulation.

### Human PSV: a Contractile Tissue

Numerous experimental studies have shown that human PSV behaved as a contractile tissue. Contractile properties of the human placenta have long been recognized. Early in the twentieth century, several authors have described smooth-like muscle cells in human PSV [22–27]. Then, it has been shown that in the presence of KCl, PSV contracted along their long axis [1,2]. Other authors [28–30] have measured the human PSV myosin content and the myosin ATPase activity (i.e., the thermodynamic flow), which represents the basic chemical characteristic of contractile tissues. The structure of the extravascular part of PSV has been described and myosin has been identified in large amount within it [31–33]. This contractile system is composed of extravascular stromal myofibroblasts arranged parallel to the PSV longitudinal axis and unlike the circular vascular smooth muscle cells [15,31,34–36]. Moreover, it has also been shown that PSV contracted under electrical stimulation in tetanus mode [16]. The myosin type found in the extra vascular part of human PSV has been shown to be the non muscle myosin IIA (NMIIA) [37], whose molecular kinetics appear to be particularly slow [38]. Finally, myosin CB kinetics in PSV have been found the slowest ever described in a human contractile tissue [3].

### Near-Equilibrium Thermodynamics

In human PSV, near-equilibrium thermodynamic behavior was validated because the chemical affinity (A) of the global CB cycle was A ≺≺ 2500 J.mol^-1^ [6,20,39] (Table 1). Both skeletal, cardiac and smooth muscles have been all reported as behaving in a near-equilibrium state [9,10]. In human PSV, the order of magnitude of affinity was moderately higher but did not greatly differ from those calculated in other contractile tissues (Tables 1 and 2). Numerous living systems have been shown to be maintained in a near-equilibrium state through a flow of energy and matter. This has been observed in chemical processes which regulate cellular metabolism and use adenosine triphosphate [40–42,43], in the Gibbs-Donnan system [44] and in the Na^+—^K^+^ ATPase pump [45].

### Linear Thermodynamics

Under linear regime where the phenomenological laws of Onsager [19] are valid, a near-equilibrium system will evolve to a stationary state [6,46]. This was the case in our study since there was a linear relationship between thermodynamic force (A/T) and thermodynamic flow (v) (Fig 2). Similar behavior has been previously described in several living systems [47]. This has been reported in mitochondria, in oxidative phosphorylation, in facilitated and active transport and in time evolution of proteins [48,49,50,51,52,53]. In such stationary open systems subject to flow of energy and matter, total entropy production rate d_i_S/dt reaches a minimum which represents the stability criterion of a stationary state [6,20]. This was the case for striated and smooth muscles [9,10]. diS/dt quantifies irreversibility of chemical processes [20]. The higher diS/dt, the further the system moves away from equilibrium [6]. The low value of entropy production rate observed in PSV (Table 1) indicated that this contractile structure generated few irreversible biochemical processes and behaved very near-equilibrium compared with other contractile systems (Table 2). This was mainly due to the dramatically low thermodynamic flow observed in PSV (Table 1). Thermodynamic flow (product of k_cat_ and myosin content) was particularly low because of the low values of both k_cat_ and myosin content in PSV (Table 1), compared to that observed in other contractile tissues (Table 2).

### Other Thermodynamic Parameters

In human PSV, statistical entropy was of the same order of magnitude (Table 1) of values as previously reported in smooth and striated muscles (Table 2) [9,10]. S is related to the dispersal of energy and represents a measure of the degree of disorder in a given system [7,54]. Microcanonical partition function (z) reflects how the total number of CB molecular motors is distributed or partitioned over the available states, and gives a rough estimate of the number of molecular states that are significantly populated at T °K. Statistical entropy, internal energy, affinity, thermodynamic force and microcanonical partition function were also of the same order of magnitude in PSV (Table 1) and in other contractile tissues (Table 2).

Our results may have some clinical interests, particularly in case of intrauterine growth restriction (IUGR), which refers to pathological states in which a fetus is unable to achieve its genetically determined potential size. IUGR which represents 3 to 5% of births, causes numerous perinatal complications, including fetal morbidity and mortality, iatrogenic prematurity, fetal compromise in labor, etc. Numerous placental abnormalities due to hypertension, pre-eclampsia or diabetes may cause IUGR. From a clinical point of view, it would be interesting to study thermodynamic properties of placenta in pathological states such as IUGR.

## Conclusions

Stem villi of human placenta were a contractile tissue containing myosin molecular motors which cycle dramatically slowly. This confers a particular thermodynamic profile, with the lowest entropy production rate ever observed in any contractile system. Moreover, human placenta behaved as a near-equilibrium system under linear regime evolving to a stationary state. The low values of both thermodynamic flow and myosin content conferred to human PSV a dramatically low rate of entropy production compared to other contractile tissues.

## References

[1] Krantz EK, Parker JC (1963) Contractile properties of the smooth muscle in the human placenta. Clin Obstet Gynecol 6: 26–38.

[2] Farley AE, Graham CH, Smith GN (2004) Contractile properties of human placental anchoring villi. Am J Physiol Regul Integr Comp Physiol 287: R680–685. 15142834 10.1152/ajpregu.00222.2004

[3] Lecarpentier Y, Claes V, Lecarpentier E, Guerin C, Hebert JL, Arsalane A et al (2014) Ultraslow myosin molecular motors of placental contractile stem villi in humans. PLoS One 9: e108814 10.1371/journal.pone.0108814 25268142 PMC4182608

[4] Huxley AF (1957) Muscle structure and theories of contraction. Prog Biophys Biophys Chem 7: 255–318. 13485191

[5] Lymn RW, Taylor EW (1971) Mechanism of adenosine triphosphate hydrolysis by actomyosin. Biochemistry 10: 4617–4624. 4258719 10.1021/bi00801a004

[6] Kondepudi D, Prigogine I (1999) Modern thermodynamics from heat engines to dissipative structures New York: Wiley & Sons 1–486 p.

[7] Atkins PW (1990) Physical Chemistry. Oxford Melbourne Tokio: Oxford University Press 1–1031 p.

[8] Levine IN (2003) Physical chemistry; McGraw-Hill, editor. New York 1–986 p.

[9] Lecarpentier Y, Blanc FX, Quillard J, Hebert JL, Krokidis X, Coirault C (2005) Statistical mechanics of myosin molecular motors in skeletal muscles. J Theor Biol 235: 381–392. 15882700 10.1016/j.jtbi.2005.01.018

[10] Lecarpentier Y, Claes V, Lecarpentier E, Blanc FX, Joseph T, Geraets B et al (2011) Comparative statistical mechanics of myosin molecular motors in rat heart, diaphragm and tracheal smooth muscle. C R Biol 334: 725–736. 10.1016/j.crvi.2011.08.001 21943522

[11] Brutsaert DL, Claes VA, Goethals MA (1973) Effect of calcium on force-velocity-length relations of heart muscle of the cat. Circ Res 32: 385–392. 4691344 10.1161/01.res.32.3.385

[12] Swynghedauw B (1999) Molecular mechanisms of myocardial remodeling. Physiol Rev 79: 215–262. 9922372 10.1152/physrev.1999.79.1.215

[13] Leguillette R, Lauzon AM (2008) Molecular mechanics of smooth muscle contractile proteins in airway hyperresponsiveness and asthma. Proc Am Thorac Soc 5: 40–46. 18094083 10.1513/pats.200704-053VS

[14] Alexander GR, Himes JH, Kaufman RB, Mor J, Kogan M (1996) A United States national reference for fetal growth. Obstet Gynecol 87: 163–168. 8559516 10.1016/0029-7844(95)00386-X

[15] Demir R, Kosanke G, Kohnen G, Kertschanska S, Kaufmann P (1997) Classification of human placental stem villi: review of structural and functional aspects. Microsc Res Tech 38: 29–41. 9260835 10.1002/(SICI)1097-0029(19970701/15)38:1/2<29::AID-JEMT5>3.0.CO;2-P

[16] Lecarpentier E, Claes V, Timbely O, Hebert JL, Arsalane A, Moumen A et al (2013) Role of both actin-myosin cross bridges and NO-cGMP pathway modulators in the contraction and relaxation of human placental stem villi. Placenta 34: 1163–1169. 10.1016/j.placenta.2013.10.007 24183754

[17] Hill AV (1938) The heat of shortening and the dynamic constants of muscle. Proc R Soc Lond Biol Sci 126: 136–195. 10.1098/rspb.1949.0019 18152150

[18] Tyska MJ, Dupuis DE, Guilford WH, Patlak JB, Waller GS, Trybus KM et al (1999) Two heads of myosin are better than one for generating force and motion. Proc Natl Acad Sci U S A 96: 4402–4407. 10200274 10.1073/pnas.96.8.4402 PMC16344

[19] Onsager L (1931) Reciprocal relations in irreversible processes II. Phys Rev 38: 405–426.

[20] Prigogine I (1967) Introduction to thermodynamics of Irreversible Processes New York: Wiley, J.

[21] De Donder T (1927) L' affinité. Paris.

[22] Happe H (1907) Beobachtungen an Eihäuten junger menschlicher Eier. Anat Hefte 32: 171.

[23] Benirschke K, Kaufmann P, Baergen RN (2006) Pathology of the Human Placenta. New York: Springer. 1069 p.

[24] Iizuka S (1916) Uber Verkammen von Muskelfasern in der menschlichen Placenta. Beitr Geburtsh Gynaek 19: 101.

[25] Naujoks H (1922) Heben anatomische Veränderungen der kindlichen Eihäute einen Einfluss auf die Zeit des Blasensprunges. Z GeburtshGynaek 84: 304.

[26] Dubreuil G, Rivière M (1932) Formations fibromusculaires du chorion et villosités du placenta humain. C R Soc Biol 111: 170–172.

[27] Spanner R (1935) Mütterlicher und kindlicher kreislauf der menschlichen Placenta und seine Strömbahnen. Z Anat Entwicklungsgesch 105: 163–242.

[28] King TM, Groeschel-Stewart U (1965) Placental Contractile Protein. Am J Obstet Gynecol 93: 253–258. 14336655 10.1016/0002-9378(65)90665-4

[29] Huszar G, Bailey P (1979) Isolation and characterization of myosin in the human term placenta. Am J Obstet Gynecol 135: 707–712. 158981 10.1016/0002-9378(79)90379-x

[30] Michael C (1974) Actomyosin content of the human placenta. J Obstet Gynaecol Br Commonw 81: 307–310. 4824689 10.1111/j.1471-0528.1974.tb00465.x

[31] Graf R, Langer JU, Schonfelder G, Oney T, Hartel-Schenk S, Reutter W et al (1994) The extravascular contractile system in the human placenta. Morphological and immunocytochemical investigations. Anat Embryol (Berl) 190: 541–548. 7534454 10.1007/BF00190104

[32] Graf R, Schonfelder G, Muhlberger M, Gutsmann M (1995) The perivascular contractile sheath of human placental stem villi: its isolation and characterization. Placenta 16: 57–66. 7716128 10.1016/0143-4004(95)90081-0

[33] Graf R, Matejevic D, Schuppan D, Neudeck H, Shakibaei M, Vetter K (1997) Molecular anatomy of the perivascular sheath in human placental stem villi: the contractile apparatus and its association to the extracellular matrix. Cell Tissue Res 290: 601–607. 9369535 10.1007/s004410050965

[34] Feller AC, Schneider H, Schmidt D, Parwaresch MR (1985) Myofibroblast as a major cellular constituent of villous stroma in human placenta. Placenta 6: 405–415. 2866507 10.1016/s0143-4004(85)80017-5

[35] Kohnen G, Castellucci M, Hsi BL, Yeh CJ, Kaufmann P (1995) The monoclonal antibody GB 42—a useful marker for the differentiation of myofibroblasts. Cell Tissue Res 281: 231–242. 7648618 10.1007/BF00583392

[36] Sparn HG, Lieder-Ochs BA, Franke WW (1994) Immunohistochemical identification and characterization of a special type of desmin-producing stromal cells in human placenta and other fetal tissues. Differentiation 56: 191–199. 8034134 10.1046/j.1432-0436.1994.5630191.x

[37] Matsumura S, Sakurai K, Shinomiya T, Fujitani N, Key K, Ohashi M (2011) Biochemical and immunohistochemical characterization of the isoforms of myosin and actin in human placenta. Placenta 32: 347–355. 10.1016/j.placenta.2011.02.008 21420731

[38] Kovacs M, Wang F, Hu A, Zhang Y, Sellers JR (2003) Functional divergence of human cytoplasmic myosin II: kinetic characterization of the non-muscle IIA isoform. J Biol Chem 278: 38132–38140. 12847096 10.1074/jbc.M305453200

[39] Prigogine I, Nicolis G, Babloyantz A (1974) Nonequilibrium problems in biological phenomena. Ann N Y Acad Sci 231: 99–105. 4522899 10.1111/j.1749-6632.1974.tb20557.x

[40] Veech RL, Lawson JW, Cornell NW, Krebs HA (1979) Cytosolic phosphorylation potential. J Biol Chem 254: 6538–6547. 36399

[41] Krebs HA, Veech RL (1969) Equilibrium relations between pyridine nucleotides and adenine nucleotides and their roles in the regulation of metabolic processes. Adv Enzyme Regul 7: 397–413. 4391643 10.1016/0065-2571(69)90030-2

[42] McGilvery RW, Murray TW (1974) Calculated equilibria of phosphocreatine and adenosine phosphates during utilization of high energy phosphate by muscle. J Biol Chem 249: 5845–5850. 4369824

[43] Marshall WE, Omachi A (1974) Measured and calculated NAD+-NADH ratios in human erythrocytes. Biochim Biophys Acta 354: 1–10. 4367846 10.1016/0304-4165(74)90046-4

[44] Masuda T, Dobson GP, Veech RL (1990) The Gibbs-Donnan near-equilibrium system of heart. J Biol Chem 265: 20321–20334. 2147022

[45] Veech RL, Kashiwaya Y, Gates DN, King MT, Clarke K (2002) The energetics of ion distribution: the origin of the resting electric potential of cells. IUBMB Life 54: 241–252. 12587974 10.1080/15216540215678

[46] Vidal C, Dewel G, Borckmans P (1994) Au-delà de l'équilibre; Hermann édseda, editor. Paris 1–372 p.

[47] Demirel Y, Sandler SI (2002) Thermodynamics and bioenergetics. Biophys Chem 97: 87–111. 12050002 10.1016/s0301-4622(02)00069-8

[48] Rigoulet M, Devin A, Espie P, Guerin B, Fontaine E, Piquet MA et al (1998) Flux-force relationships in intact cells: a helpful tool for understanding the mechanism of oxidative phosphorylation alterations? Biochim Biophys Acta 1365: 117–124. 9693730 10.1016/s0005-2728(98)00051-6

[49] Stucki JW (1980) The optimal efficiency and the economic degrees of coupling of oxidative phosphorylation. Eur J Biochem 109: 269–283. 7408881 10.1111/j.1432-1033.1980.tb04792.x

[50] Stucki JW (1991) Non-equilibrium thermodynamic sensitivity of oxidative phosphorylation. Proc R Soc Lond B Biol Sci 244: 197–202. 10.1098/rspb.1991.0070 1679938

[51] Kedem O, Katchalsky A (1989) Thermodynamic analysis of the permeability of biological membranes to non-electrolytes. 1958. Biochim Biophys Acta 1000: 413–430. 2673395

[52] Caplan SR, Essig A (1969) Oxidative phosphorylation: thermodynamic criteria for the chemical and chemiosmotic hypotheses. Proc Natl Acad Sci U S A 64: 211–218. 5263005 10.1073/pnas.64.1.211 PMC286149

[53] Dewey TG, Delle Donne M (1998) Non-equilibrium thermodynamics of molecular evolution. J Theor Biol 193: 593–599. 9745755 10.1006/jtbi.1998.0724

[54] Prigogine I, Nicolis G (1971) Biological order, structure and instabilities. Q Rev Biophys 4: 107–148. 4257403 10.1017/s0033583500000615

